# Understanding Childbirth Perceptions Among Healthcare Providers: A Cross-Sectional Study Using the Birth Beliefs Scale

**DOI:** 10.7759/cureus.109933

**Published:** 2026-05-30

**Authors:** Maimoona Ahmed, Pallavi Chandra Ravula

**Affiliations:** 1 Obstetrics and Gynecology, Fernandez Foundation, Hyderabad, IND

**Keywords:** attitude, beliefs, childbirth, healthcare provider, survey

## Abstract

Aim

Healthcare providers’ beliefs about childbirth, as a natural or medical process, shape labor management and women’s birthing experiences. This study aimed to objectively assess providers’ attitudes using the Birth Beliefs Scale (BBS).

Materials and methods

A cross-sectional online survey was conducted during a maternity-care conference at a tertiary center in November 2025. Participants completed a web-based questionnaire based on the BBS. The survey assessed beliefs about birth as a natural process, such as seeing birth as normal, safe, and something women’s bodies are designed for, emphasizing trust in the body, acceptance of pain as intrinsic, and minimal intervention unless necessary. Questions on beliefs about birth as a medical process covered perceptions of childbirth as dangerous and risky, safe only in hindsight, requiring medical supervision, and viewing labor pain as unnecessary and manageable pharmacologically. Independent t-tests compared continuous variables between two groups, while one-way ANOVA with Bonferroni post-hoc tests assessed differences across multiple groups. Ordinal alpha coefficients evaluated the internal consistency of both BBS subscales, with statistical significance set at p < 0.05. Given the ordinal nature of Likert-scale items, the ordinal alpha coefficient was employed to assess internal consistency and reliability of the two BBS subscales within this study population, accounting for potential differences from the original validation context.

Results

Of the 178 respondents, 56 (31.5%) were obstetricians, 52 (29.2%) midwives, 18 (10.1%) nurses, 12 (6.7%) childbirth educators, 11 (6.2%) doulas, and 29 (6.3%) others. Among the respondents, 104 (58.4%) worked in the private sector. Overall, BBS-natural scores were high. However, BBS-medical scores differed significantly by age, profession, and sector. Younger providers, obstetricians, and private-sector clinicians showed stronger beliefs favoring medical intervention during childbirth.

Conclusion

Participants largely endorsed naturalistic views of birth, but attitudes varied substantially across provider groups, age brackets, and practice settings. These differences reflect the influence of training and institutional culture on childbirth perceptions. Targeted inter-professional education, supportive institutional policies, and strengthened midwifery models may help promote balanced, evidence-based, woman-centered maternity care in India.

## Introduction

Pregnancy and childbirth are profound experiences in a woman’s life that shape her transition into motherhood. For most women, childbirth is a normal, healthy event with the potential to be a deeply empowering experience [1]. However, a significant gap exists between the best available evidence in maternal health and routine clinical practices. Birth has increasingly become medicalized, with tendencies toward elective induction of labor or planned caesarean section on request, active management to accelerate labor, and reliance on regional anesthesia for pain relief. These interventions have not been shown to consistently improve birth outcomes, and in some cases, such as unindicated caesarean sections, they may even cause serious harm [2].

Researchers have found that lay support during labor is associated with fewer interventions, lower caesarean rates, and reduced use of oxytocin and labor analgesia, compared with professional labor support [3]. Other studies have demonstrated that women’s birthing experiences are shaped more by their interactions with healthcare providers than by the specific medical interventions or type of birth they undergo [4]. Concerns have also been raised that many women do not have adequate opportunities to make informed choices about the type of birth that best suits their needs, and those who do have choices may place excessive, and often uninformed, reliance on their doctors [5]. This suggests that the type of healthcare provider influences birth management and, consequently, the birthing experience. Providers’ interactions with patients are shaped by personal factors as well as broader social and systemic influences. Their perspectives are key to identifying barriers and developing interventions that support evidence-based, respectful maternity care [6].

Assessment of provider attitudes toward birth has previously been conducted using the Birth Beliefs Scale (BBS), originally developed to measure perceptions of childbirth as a natural versus a medical process among pregnant women [7,8]. The beliefs about birth as a natural process imply ideals such as birth is a normal and safe process, women’s bodies are designed to give birth, women should trust their bodies, pain is an intrinsic part of the birthing process, and there should be no interference in birth unless necessary. The beliefs about birth as a medical process imply such ideals as birth is dangerous and risky, it can only be considered safe in hindsight, it should be performed under the vigilant care of a trained medical professional, and labor pain is an unnecessary inconvenience that should be taken care of pharmacologically [7].

To date, no studies have applied the BBS to healthcare providers in India, and no cross-sectional research has examined how different cadres within the Indian maternity-care system conceptualize birth. The Indian maternal healthcare workforce is uniquely heterogeneous, comprising obstetricians, midwives, nurses, doulas, and childbirth educators [9]. Understanding birth beliefs across this diverse professional spectrum is crucial in a setting where maternity-care practices range from highly medicalized environments to emerging midwifery-led models of care.

With this background, we conducted a cross-sectional study to explore healthcare providers’ perceptions of childbirth using the BBS.

## Materials and methods

Study design and setting

A cross-sectional, survey-based study was conducted during a national conference organized by our tertiary care maternity center in November 2025. The conference brought together a diverse group of participants involved in maternal and perinatal health, including doctors, midwives, nurses, childbirth educators, physiotherapists, and researchers. Attendees represented both public and private healthcare sectors across various states of India, ensuring heterogeneity in professional backgrounds and regional perspectives. The study aimed to assess participants’ beliefs about childbirth using the BBS [7]. The survey was administered online through a secure link shared with attendees during the conference.

Instrument and questionnaire design

The questionnaire (see Appendices) was adapted from the original BBS [7] and structured into three sections. The first section covered the demographic profile and included questions on age, gender, profession, years of experience, and type of institution (public or private). The second part covered beliefs about birth as a medical process and contained items reflecting perceptions of childbirth primarily as a biomedical or intervention-dependent process. The third section covered beliefs about birth as a natural process and included statements emphasizing childbirth as a natural, physiological event.

Data collection procedure

The survey was distributed to all conference attendees via an online link. Participation was voluntary, and implied consent was obtained through the completion of the questionnaire. No personally identifiable information was collected, ensuring anonymity and confidentiality of responses.

Statistical analysis

All responses were exported into Microsoft Excel 2021 (Microsoft Corporation, Redmond, WA, USA) for initial data cleaning and coding. Statistical analyses were performed using RStudio (Posit, Boston, MA, USA, http://www.posit.co).

Descriptive statistics were used to summarize the data. Categorical variables were expressed as frequencies and percentages. Continuous and ordinal variables were presented as mean ± standard deviation (SD).

Group comparisons were performed using independent t-tests for two-group comparisons and one-way analysis of variance (ANOVA) with Bonferroni post-hoc correction for comparisons involving more than two groups.

Given the ordinal nature of Likert-scale items, the ordinal alpha coefficient was employed to assess internal consistency and reliability of the two BBS subscales within this study population, accounting for potential differences from the original validation context.

A p-value <0.05 was considered statistically significant for all analyses.

Ethical considerations

Ethical approval for the study was obtained from the Institutional Review Board, Fernandez Hospital Educational & Research Foundation, Hyderabad (IRB Approval No.: 59_2025, dated November 14, 2025). Participation was voluntary, informed consent was implied, and no financial or non-financial incentives were offered to participants.

## Results

A total of 178 attendees responded to the questionnaire. Table 1 details the demographic composition of the respondents.

**Table 1:** Baseline characteristics of the study population (N=178) SD: standard deviation; NGO: non-governmental organization

Variables	Summary statistics
Age (years), Mean ± SD	38.31 ± 9.78
Age group, n (%)	
21 to 30 years	46 (25.8%)
31 to 45 years	99 (55.6%)
>45 years	33 (18.5%)
Healthcare provider, n (%)	
Obstetrician	56 (31.5%)
Midwife	52 (29.2%)
Nurse	18 (10.1%)
Childbirth educator	12 (6.7%)
Doula	11 (6.2%)
Others	29 (16.3%)
Healthcare sector, n (%)	
Private	104 (58.4%)
Public	62 (34.8%)
NGO	12 (6.7%)

The second and third sections of the survey questionnaire covered the items of the BBS. These were divided into BBS-Med for items representing the medical process and BBS-Nat for those representing the natural process. Table 2 lists the scores for both.

**Table 2:** Birth Beliefs Scale in the study population (N=178) SD: standard deviation; BBS-Med: Birth Beliefs Scale - medical; BBD-Nat: Birth Beliefs Scale - natural

Scale items	Mean ± SD	Strongly agree	Agree	Neutral	Disagree	Strongly disagree
BBS-Med						
Childbirth requires vigilant medical supervision	3.4 ± 1.3	40 (22.5%)	61 (34.3%)	27 (15.2%)	31 (17.4%)	19 (10.7%)
There are many things that can go wrong during childbirth	3.43 ± 1.25	35 (19.7%)	71 (39.9%)	23 (12.9%)	33 (18.5%)	16 (9%)
Often, a woman’s body structure does not allow her to give birth naturally	1.89 ± 1.19	9 (5.1%)	18 (10.1%)	8 (4.5%)	52 (29.2%)	91 (51.1%)
Birth is a medical event	1.89 ± 1.08	3 (1.7%)	19 (10.7%)	19 (10.7%)	51 (28.7%)	86 (48.3%)
Childbirth is a dangerous process	1.58 ± 0.91	3 (1.7%)	8 (4.5%)	9 (5.1%)	50 (28.1%)	108 (60.7%)
Nowadays, there is no reason why women should suffer pain in childbirth	2.68 ± 1.42	25 (14%)	33 (18.5%)	30 (16.9%)	40 (22.5%)	50 (28.1%)
BBS-Nat						
Birth is an empowering experience	4.61 ± 0.77	127 (71.3%)	42 (23.6%)	4 (2.2%)	1 (0.6%)	4 (2.2%)
Pain in childbirth is a significant part of the birth experience	3.72 ± 1.12	42 (23.6%)	84 (47.2%)	23 (12.9%)	18 (10.1%)	11 (6.2%)
Birth is a natural event	4.66 ± 0.66	129 (72.5%)	44 (24.7%)	1 (0.6%)	2 (1.1%)	2 (1.1%)
A woman’s body knows how to give birth	4.52 ± 0.75	111 (62.4%)	54 (30.3%)	9 (5.1%)	2 (1.1%)	2 (1.1%)
Labor should be allowed to proceed at its own pace	4.46 ± 0.82	106 (59.6%)	56 (31.5%)	10 (5.6%)	3 (1.7%)	3 (1.7%)

The BBS-Med score ranged from 1 to 4.33 with a mean (±SD) of 2.48 (±0.72), and the BBS-Nat score ranged from 1.2 to 5 with a mean (±SD) of 4.39 (±0.57). Thus, there was more agreement towards natural birth beliefs in the cohort overall.

A sub-analysis was conducted to compare the BBS scores with the type of healthcare provider, type of organization, and age group to check for any significant difference in the scores.

Table 3 depicts the comparison of the BBS-Med scores with the demographic characteristics.

**Table 3:** Comparison of BBS-Med scores according to baseline characteristics Data are presented as mean ± SD. P-values were calculated using one-way ANOVA for variables with more than two categories (Age group, Healthcare provider) and an independent sample t-test for a dichotomous variable (Healthcare sector). Test statistics are reported as F-values for ANOVA and t-values for t-tests. A p-value < 0.05 was considered statistically significant. Statistically significant p-values are denoted by ‘’*”. SD: standard deviation; BBS-Med: Birth Beliefs Scale - medical

Variables	BBS-Med, Mean ± SD	Test statistic	P-value
Age group			
21 to 30 years	2.66 ± 0.68	F = 6.253	0.002*
31 to 45 years	2.52 ± 0.76		
>45 years	2.11 ± 0.52		
Healthcare provider			
Obstetrician	2.86 ± 0.75	F = 13.688	<0.001*
Midwife	2.06 ± 0.46		
Nurse	2.96 ± 0.70		
Childbirth educator	2.11 ± 0.61		
Doula	1.92 ± 0.43		
Others	2.55 ± 0.60		
Healthcare sector			
Private	2.58 ± 0.74	t = 2.172	0.031*
Public/NGO	2.34 ± 0.68		

There was a statistically significant difference in mean BBS-Med scores across age groups (p = 0.002). Bonferroni post-hoc comparisons showed that individuals aged >45 years had significantly lower BBS-Med scores than those aged 21 to 30 years and 31 to 45 years. Mean BBS-Med scores also differed significantly by type of healthcare provider (p < 0.001); nurses and obstetricians had significantly higher scores compared with midwives, childbirth educators, and doulas. Additionally, healthcare providers from the private sector had significantly higher BBS-Med scores than those from the public/NGO sector (p = 0.031).

Table 4 depicts the comparison of the BBS-Nat scores with the demographic characteristics.

**Table 4:** Comparison of BBS-Nat scores according to baseline characteristics Data are presented as mean ± SD. P-values were calculated using one-way ANOVA for variables with more than two categories (Age group, Healthcare provider) and an independent sample t-test for a dichotomous variable (Healthcare sector). Test statistics are reported as F-values for ANOVA and t-values for t-tests. A p-value < 0.05 was considered statistically significant. Statistically significant p-values are denoted by ‘’*”. SD: standard deviation; BBD-Nat: Birth Beliefs Scale - natural

Variables	BBS-Nat, Mean ± SD	Test statistic	P-value
Age group			
21 to 30 years	4.42 ± 0.39	F = 0.094	0.910
31 to 45 years	4.38 ± 0.66		
>45 years	4.40 ± 0.52		
Healthcare provider			
Obstetrician	4.14 ± 0.61	F = 4.08	0.002*
Midwife	4.49 ± 0.62		
Nurse	4.46 ± 0.45		
Childbirth educator	4.60 ± 0.40		
Doula	4.75 ± 0.36		
Others	4.45 ± 0.45		
Healthcare sector			
Private	4.38 ± 0.47	t = 0.397	0.692
Public/NGO	4.41 ± 0.70		

There was no statistically significant difference in mean BBS-Nat scores across age groups or healthcare sectors (p > 0.05). However, mean BBS-Nat scores differed significantly by healthcare provider type (p = 0.002); Bonferroni post-hoc analysis showed that obstetricians had significantly lower BBS-Nat scores compared with doulas and midwives.

Since our study setting differed from the original validation setting for the BBS, ordinal alpha was used to assess internal consistency of both subscales of the BBS due to the ordinal (Likert-scale) nature of the items, and to confirm reliability. The BBS-Med subscale showed acceptable internal consistency (ordinal α = 0.743), while the BBS-Nat subscale demonstrated good internal consistency (ordinal α = 0.826).

## Discussion

In our cross-sectional study of 178 healthcare providers from diverse professional backgrounds across India, overall beliefs regarding childbirth were strongly aligned with the concept of birth as a natural process, as reflected by the high mean BBS-Nat score (4.39 ± 0.57). In contrast, the mean BBS-Med score (2.48 ± 0.72) indicated comparatively lower agreement with medicalized perceptions of childbirth. These findings are consistent with earlier work using the BBS, which demonstrated that healthcare professionals and pregnant individuals tend to endorse naturalistic beliefs more strongly than medicalized ones [7]. There are, however, differences in the beliefs depending on training and professional culture.

Interpretation of differences in medicalized beliefs

We found significant differences in BBS-Med scores across age groups, which suggests that older providers (>45 years) may hold less medicalized views of childbirth compared with their younger counterparts. Greater clinical experience in physiological birth in earlier eras of practice could partly explain this pattern. Similar age-related trends, where senior clinicians express more confidence in the normalcy of labor, have been noted in other maternity-care studies [10].

Profession-related differences in BBS-Med scores were also pronounced. Obstetricians and nurses demonstrated significantly higher medicalized beliefs as compared with midwives, childbirth educators, and doulas. This aligns with established literature showing that obstetric training, which focuses on risk detection and clinical intervention, often shapes more medicalized attitudes toward childbirth [11,12]. In contrast, midwives and doulas typically receive training grounded in physiology, continuity of care, and minimal intervention [13], which likely explains the lower BBS-Med scores observed in these groups. Such differences have been reported across multiple health systems, including in India, where models of care remain highly obstetric-centric [14].

Our study also highlighted that healthcare providers from the private sector endorsed more medicalized beliefs than those from public/NGO settings. This finding mirrors global patterns in which private-sector maternity care is associated with higher intervention rates, increased liability concerns, and institutional norms favoring technological surveillance [15]. This raises important considerations for ongoing efforts to reduce unnecessary interventions and promote respectful maternity care within India’s mixed health-care environment.

Interpretation of differences in naturalistic beliefs

Overall, the BBS-Nat scores were high across the cohort, but obstetricians scored significantly lower than midwives and doulas. This again reflects the influence of professional training environments and philosophical orientations of different cadres. Research also shows that even if obstetricians acknowledge the natural aspects of birth, they counter it with a heightened awareness of potential complications [16]. Midwives, on the other hand, tend to promote physiological processes and women’s innate birthing capacity [13].

We did not find any significant differences in BBS-Nat scores by age group or healthcare sector. This suggests that naturalistic beliefs may be more consistently held across demographic backgrounds, possibly reflecting widespread adoption of global narratives promoting normal birth, empowerment, and respectful maternity care [17].

Reliability of the BBS in the Indian context

The ordinal alpha values obtained in this study (0.743 for BBS-Med and 0.826 for BBS-Nat) indicate acceptable to good internal consistency within this Indian provider cohort, consistent with the reliability reported in the original validation study by Preis et al. [7]. This strengthens the applicability of the BBS in diverse cultural and professional contexts and supports its continued use for research and training evaluation in India.

Implications for practice, training, and policy

Understanding childbirth beliefs among healthcare providers is crucial because provider attitudes influence communication with women, labor management practices, and intervention rates [12,14]. The notable difference in beliefs between obstetricians and midwifery philosophy aligned professions underscores the need for inter-professional dialogue and collaborative training models that balance risk management with respect for physiological birth. Incorporating evidence-based normal birth modules, simulation exercises, and Respectful Maternity Care (RMC) frameworks into obstetric and nursing curricula could help narrow these philosophical divides between the healthcare providers.

Findings related to private-sector providers highlight an area of strategic importance for policymakers, given India’s high reliance on private maternity care. Developing supportive clinical guidelines, monitoring intervention rates, and promoting midwife-led continuity models could help address medicalized practice norms and reduce unnecessary interventions.

Strengths and limitations

A key strength of this study is the inclusion of a wide range of provider types from multiple Indian states, enhancing representativeness. The use of the validated BBS adds methodological robustness. However, findings should be interpreted considering several limitations. The convenience sampling of conference attendees may introduce selection bias, as participants might already be interested in evidence-based or physiological approaches to childbirth. The self-reported beliefs may not accurately reflect actual clinical practice. Further research should include larger, multi-site samples and examine correlations between provider beliefs and observed labor management behavior.

## Conclusions

Understanding childbirth beliefs among healthcare providers is crucial as provider attitudes influence communication with the patient, labor management practices, and intervention rates. Healthcare providers in our study generally endorsed naturalistic beliefs about childbirth, though significant variation existed by profession, age, and healthcare sector. These patterns highlight the influence of training and institutional culture on childbirth perceptions. Addressing these differences through inter-professional education, institutional reform, joint maternity care provider conferences, and expanded midwifery models may support more balanced, evidence-based, woman-centered maternity care in India.

## Appendices

Birth Beliefs Scale survey

You are invited to take part in this survey aimed at understanding individuals’ beliefs and perceptions about childbirth. The information collected through the Birth Beliefs Scale will help researchers gain insights into attitudes, fears, and confidence related to childbirth. Your participation in this study is entirely voluntary. You may choose to stop or skip any question at any time without any penalty or negative consequence. All responses will be kept strictly confidential. The data will be used only for research purposes.

Consent Statement:

By proceeding to answer the Birth Beliefs Scale questionnaire, you acknowledge that: You have read and understood the above information and are voluntarily agreeing to participate in this study.

* Indicates required question

Demographics

Age*

Healthcare Provider Category*

Healthcare Sector*

Belief about birth as a medical process (BBS-Med)

1.     Childbirth requires vigilant medical supervision*

Strongly Disagree

Disagree

Neutral

Agree

Strongly Agree

2.     There are many things that can go wrong during childbirth*

Strongly Disagree

Disagree

Neutral

Agree

Strongly Agree

3.     Often, a woman’s body structure does not allow her to give birth naturally*

Strongly Disagree

Disagree

Neutral

Agree

Strongly Agree

4.     Birth is a medical event*

Strongly Disagree

Disagree

Neutral

Agree

Strongly Agree

5.     Childbirth is a dangerous process*

Strongly Disagree

Disagree

Neutral

Agree

Strongly Agree

6.     Nowadays, there is no reason why women should suffer pain in childbirth*

Strongly Disagree

Disagree

Neutral

Agree

Strongly Agree

Belief about birth as a natural process

1.     Birth is an empowering experience*

Strongly Disagree

Disagree

Neutral

Agree

Strongly Agree

2.     Pain in childbirth is a significant part of the birth experience*

Strongly Disagree

Disagree

Neutral

Agree

Strongly Agree

3.     Birth is a natural event*

Strongly Disagree

Disagree

Neutral

Agree

Strongly Agree

4.     A woman’s body knows how to give birth*

Strongly Disagree

Disagree

Neutral

Agree

Strongly Agree

5.     Labor should be allowed to proceed at its own pace*

Strongly Disagree

Disagree

Neutral

Agree

Strongly Agree
